# Unlocking the Antioxidant Potential of Pigeon Peas (*Cajanus cajan* L.) via Wild Fermentation and Extraction Optimization

**DOI:** 10.3390/foods15020310

**Published:** 2026-01-15

**Authors:** Tamara Machinjili, Chikondi Maluwa, Chawanluk Raungsri, Hataichanok Chuljerm, Pavalee Chompoorat Tridtitanakiat, Elsa Maria Salvador, Kanokwan Kulprachakarn

**Affiliations:** 1 Department of Chemical Engineering, Faculty of Engineering, Eduardo Mondlane University, Maputo 3453, Mozambique; tamarachirambo.tc@gmail.com; 2 Department of Community Development, Ministry of Gender, Community Development and Social Welfare, Lilongwe Private Bag 330, Malawi; 3 School of Health Sciences Research, Research Institute for Health Sciences, Chiang Mai University, Chiang Mai 50200, Thailand; chikondi_maluwa@cmu.ac.th (C.M.); hataichanok.ch@cmu.ac.th (H.C.); 4 Faculty of Clinical Sciences, Malawi College of Health Sciences, Blantyre Campus, Blantyre Private Bag 396, Malawi; 5 Division of Product Development Technology, Faculty of Agro-Industry, Chiang Mai University, Chiang Mai 50100, Thailand; chawanlukaung@gmail.com (C.R.); pavalee.t@cmu.ac.th (P.C.T.); 6 Department of Biological Sciences, Faculty of Sciences, Eduardo Mondlane University, Maputo 3453, Mozambique; elsamariasalvador@gmail.com

**Keywords:** DPPH, ABTS, FRAP, phenolic compounds, functional foods, bioactive compounds, legume processing, pigeon peas

## Abstract

Oxidative stress contributes significantly to chronic disease burden, necessitating identification of accessible dietary antioxidant sources. Pigeon peas (*Cajanus cajan* L.) contain substantial bioactive compounds, yet most exist in bound forms with limited bioavailability. This study evaluated wild fermentation combined with systematic extraction optimization to enhance antioxidant recovery from pigeon peas. Seeds underwent wild fermentation in brine solution, followed by extraction under varying conditions (seven solvent systems, three temperatures, and three-time durations). Multiple complementary assays assessed antioxidant capacity (total phenolic content, DPPH radical scavenging, ferric reducing power, and ABTS activity). Fermentation substantially improved antioxidant properties across all parameters, with particularly pronounced effects on radical scavenging activities. Extraction optimization identified 70% methanol at 40 °C for 24 h as optimal, demonstrating marked improvements over conventional protocols. Strong intercorrelations among assays confirmed coordinated enhancement of multiple antioxidant mechanisms rather than isolated changes. The findings demonstrate that both biotechnological processing and analytical methodology critically influence antioxidant characterization in pigeon peas. This integrated approach offers practical guidance for developing antioxidant-rich functional foods, particularly relevant for resource-limited settings where pigeon peas serve as dietary staples. The study establishes foundation for translating fermentation technology into nutritional interventions, though further research addressing bioavailability, microbiological characterization, and bioactive compound identification remains essential.

## 1. Introduction

Oxidative stress, characterized by an imbalance between reactive oxygen species (ROS) production and antioxidant defense mechanisms, plays a big role in the pathogenesis of numerous chronic diseases, including cardiovascular disorders, diabetes, cancer, chronic respiratory diseases, and neurodegenerative diseases [1,2,3]. Hence, there has been increasing interest in natural antioxidants derived from plant sources as alternatives to synthetic compounds, which are very expensive and may pose potential health risks [4,5].

Legumes, particularly pigeon peas (*Cajanus cajan* L.), represent a promising source of bioactive compounds with significant antioxidant potential [6]. Pigeon peas constitute one of the most important grain legumes in tropical and subtropical regions, ranking sixth in global legume production with approximately 4.9 million tons produced annually [7]. Beyond their nutritional significance as a source of protein, complex carbohydrates, and essential minerals, pigeon peas contain substantial amounts of bioactive compounds, including phenolic acids, flavonoids, anthocyanins, and other phytochemicals that contribute to their antioxidant properties [8,9]. However, many of these bioactive compounds exist in bound forms or within cellular matrices that limit their bioavailability and biological activity [10].

Fermentation, an ancient food processing technique, offers a sustainable biotechnological approach to enhance the functional properties of plant-based foods [11]. During fermentation, endogenous and microbial enzymes catalyze complex biochemical transformations that can significantly alter the composition and bioactivity of food matrices [12]. These enzymatic processes include the hydrolysis of glycosidic bonds in phenolic compounds, the breakdown of cell wall structures that release bound antioxidants, and the synthesis of novel bioactive metabolites through microbial metabolism [13,14], increasing free phenolic compounds by 50–300% [15,16].

Previous studies have demonstrated that fermentation can substantially enhance the antioxidant properties of various legumes and cereals. For instance, fermentation of cowpeas with *Lactobacillus plantarum* resulted in significant increases in free phenolic content and radical scavenging activity [17]. Similarly, fermented black beans showed enhanced DPPH and ABTS radical scavenging activities compared to their unfermented counterparts [18]. However, the effects of fermentation on antioxidant properties appear to be species-specific and dependent on fermentation conditions, microbial strains, and duration [19]. Solid-state fermentation with lactic acid bacteria (LAB) like *Pediococcus pentosaceus* utilizes high enzyme activity (0.67 U/g β-glucosidase), validated by FTIR/SEM structural changes [15].

The complexity of antioxidant systems necessitates the use of multiple assays to comprehensively evaluate antioxidant capacity, as different methods measure distinct aspects of antioxidant activity [20]. The DPPH (2,2-diphenyl-1-picrylhydrazyl) assay evaluates the ability to scavenge stable free radicals, while the ABTS [2,2′-azinobis-(3-ethylbenzothiazoline-6-sulfonic acid)] assay measures the capacity to neutralize cationic radicals [21]. The FRAP (Ferric Reducing Antioxidant Power) assay quantifies the reducing capacity of antioxidants by measuring their ability to reduce ferric ions (Fe^3+^) to ferrous ions (Fe^2+^) [22]. Total phenolic content (TPC) provides an estimate of the overall phenolic compounds present, which are major contributors to antioxidant activity in plant materials [23]. Using multiple essays in determining the antioxidant activity is crucial for explaining the biochemical basis of antioxidant activity and predicting their functional properties.

Beyond fermentation, extraction methodology plays a critical role in the recovery and quantification of antioxidant compounds from plant materials. Extraction efficiency is influenced by multiple factors including solvent polarity, temperature, time, and interactions [24]. Despite the importance of extraction optimization, most studies on fermented legumes have employed single, non-optimized extraction protocols, potentially underestimating the true antioxidant potential of these materials [25].

Despite pigeon peas being a nutritional staple in resource-limited settings, their antioxidant potential remains underutilized since most bioactive compounds exist in bound, poorly bioavailable forms within the seed matrix [6]. Furthermore, standard extraction protocols fail to adequately recover these compounds, leading to significant underestimation of their true functional value. These limitations restrict both scientific understanding and the practical application of pigeon peas as functional food ingredients.

While individual studies have examined either fermentation effects or extraction optimization in legumes, no systematic investigation has evaluated their combined, potentially synergistic effects on pigeon pea antioxidants. Existing fermentation studies on pigeon peas have employed single extraction protocols without systematic optimization, potentially masking the true effects of fermentation-induced changes [26,27].

Therefore, this study aimed to evaluate the combined effects of wild fermentation and optimized extraction conditions on the antioxidant properties of pigeon peas using multiple complementary antioxidant assays, including TPC, DPPH, FRAP, and ABTS. Specifically, the study aimed to (i) determine the effect of spontaneous fermentation on antioxidant activity and (ii) identify optimal solvent composition, temperature, and extraction time for maximizing antioxidant recovery. We hypothesize that wild fermentation will significantly enhance the antioxidant capacity of pigeon peas through the enzymatic liberation and biotransformation of bound phenolic compounds, and that systematic optimization of extraction parameters will further maximize the recovery of both native and fermentation-generated antioxidants.

The findings of this research contribute to the scientific understanding of fermentation-induced changes in legume antioxidant systems while providing practical guidance for optimal extraction protocols. Additionally, this work demonstrates the importance of employing both biotechnological processing and optimized analytical approaches to appreciate the complex effects of food processing on bioactive compound profiles and their functional properties.

## 2. Materials and Methods

### 2.1. Sample Preparation

Dried pigeon peas were procured from local agricultural markets in Maputo, Mozambique and authenticated by the Agricultural Research Institute of Mozambique, Maputo, Mozambique. Seeds were cleaned, sorted, and divided into two groups: unfermented controls and fermentation samples. For fermentation, wild fermentation was used as described by Fleming et al. [28] with some modifications. Cleaned pigeon peas were fully immersed in a plastic bucket of 10% brine solution (100 g of locally purchased unionized salt in 1 L of water with no chlorine) and were covered with a cotton cloth. The bucket was then left in a dark room for 96 h at ambient temperature. Fermented samples were then sun-dried for 48 h. Both fermented and unfermented samples were ground into fine powder (60-mesh size) using a laboratory hammer mill (Model LM 3100, PerkinElmer, Stockholm, Sweden) and stored in high-density polyethylene (HDPE) (0.77 mm thick) bags until analysis. The analysis of the samples was done at the faculty of Agro-Industry laboratories, Chiang Mai University, Chiang Mai, Thailand. The replicates were categorized into biological replicates representing independent pigeon pea batches processed separately (fermented vs. unfermented) and technical replicates which were replicate extractions/antioxidant assays from each biological sample.

### 2.2. Optimization of Extraction Conditions

The systematic optimization of extraction parameters followed a one-factor-at-a-time (OFAT) approach as described by [29], conducted to determine the most effective extraction parameters for recovering antioxidant compounds from both fermented and unfermented pigeon peas. This sequential optimization strategy evaluates the effect of individual factors while maintaining other variables constant, allowing identification of optimal conditions for each parameter. The optimization involved three main factors: solvent type/concentration, extraction temperature, and extraction time.

OFAT optimization strategy was used instead of multivariate approaches such as response surface methodology (RSM) or factorial designs simply because it allows efficient evaluation of the main effects of individual extraction parameters. Additionally, this approach provides simple, actionable guidance suitable for resource-limited settings where pigeon peas are commonly consumed.

#### 2.2.1. Solvent Selection Study

Ground samples (10 g) were extracted with 100 mL of different solvent systems to evaluate the effect of solvent polarity on antioxidant recovery. The following solvents were tested: (1) Methanol (ACS Reagent grade, 99.8% Sigma-Aldrich, St. Louis, MO, USA) at three concentrations: 50%, 70%, and 80% (*v*/*v* in distilled water); (2) Ethanol (ACS Reagent grade, 99.8% Fischer Scientific, Loughborough, UK) at three concentrations: 50%, 70%, and 80% (*v*/*v* in distilled water); (3) Distilled water (100%). All extractions in the solvent optimization phase were performed at room temperature (25 °C) for 24 h with continuous shaking at 150 rpm using an orbital shaker.

#### 2.2.2. Temperature Optimization Study

Using the optimal solvent concentration identified from the solvent selection study (70% methanol), extractions were performed at three different temperatures to evaluate the effect of thermal energy on extraction efficiency: (1) 25 °C (room temperature, control condition); (2) 40 °C (moderate heating); (3) 60 °C (elevated temperature). Temperature-controlled water baths were used to maintain constant temperatures. Extraction duration was maintained at 24 h with continuous shaking at 150 rpm.

#### 2.2.3. Time-Course Extraction Study

To determine the optimal extraction duration, time-course experiments were conducted using the optimal solvent (70% methanol) at the optimal temperature (40 °C) identified from previous optimization steps. Extractions were performed for three different time durations: (1) 12 h; (2) 24 h; (3) 48 h. All course extractions were conducted with continuous shaking at 150 rpm.

### 2.3. Sample Extraction

All extracts, regardless of extraction conditions, were processed identically after the extraction period. Samples were filtered with 13 mm Nylon Syringe Filter 0.45 µm with Outer Ring (Zhejiang ALWSCI Technologies Co., Ltd., Shaoxing, China). The solvents were removed by evaporation at room temperature in a fume hood. The resulting extracts were stored at 4 °C for later analysis, which was performed within 48 h of extraction to minimize degradation.

### 2.4. Antioxidant Activity Assays

#### 2.4.1. Total Phenolic Content (TPC)

Total phenolic content was determined using the Folin–Ciocalteu colorimetric method [30] with some modifications. Briefly, 0.5 mL of extract was mixed with 2.5 mL of 10% Folin–Ciocalteu reagent and allowed to react for 5 min. Subsequently, 2 mL of 7.5% sodium carbonate solution was added, and the mixture was incubated in the dark at room temperature for 90 min. Absorbance was measured at 765 nm in a UV–visible spectrophotometer (Tecan Spark Cyto wins, Maennedorf, Switzerland), and results were expressed as milligrams of gallic acid equivalents per gram of dry weight (mg GAE/g DW). The blank was prepared by substituting the same amount of diluted extract with solvent.

#### 2.4.2. DPPH Radical Scavenging Activity

The DPPH radical scavenging activity was determined according to the method used by Chaves [31] with minor modifications. Various concentrations of sample extracts (0.1, 1, and 2.0 mg/mL) were mixed with 2 mL of 0.1 mM DPPH solution in solvent. The mixture was incubated in darkness for 30 min at room temperature, and absorbance was measured at 517 nm using a UV–visible spectrophotometer (Tecan Spark Cyto wins, Switzerland). Results were expressed as IC_50_ (half maximal inhibitory concentration) values in milligrams per milliliter (mg/mL), representing the concentration required to scavenge 50% of DPPH radicals. The blank was prepared with the solvent dilution of DPPH.

#### 2.4.3. Ferric Reducing Antioxidant Power (FRAP)

The FRAP assay was performed according to [32] with some modifications. The FRAP reagent was prepared by mixing 300 mM acetate buffer (pH 3.6), 10 mM TPTZ (2,4,6-tripyridyl-s-triazine) solution, and 20 mM ferric chloride solution in a 10:1:1 ratio. Sample extract (100 μL) was mixed with 3 mL of FRAP reagent and incubated at 37 °C for 10 min. Absorbance was measured at 593 nm using a UV–visible spectrophotometer (Tecan Spark Cyto wins, Switzerland), and results were expressed as micromoles of Trolox equivalents per gram of dry weight (μmol TE/g DW). The blank was prepared by substituting the same amount of diluted extract with solvent.

#### 2.4.4. ABTS Radical Cation Decolorization Assay

The ABTS assay was conducted following the description of [33] with modifications. The ABTS radical cation (ABTS^•+^) was generated by mixing 7 mM ABTS stock solution with 2.45 mM potassium persulfate and allowing the mixture to stand in darkness for 16 h. The ABTS^•+^ solution was diluted with phosphate buffer (pH 7.4) to achieve an absorbance of 0.70 ± 0.02 at 734 nm. Sample extract (100 μL) was mixed with 1 mL of diluted ABTS^•+^ solution, and absorbance was measured at 734 nm after 6 min of incubation using a UV–visible spectrophotometer (Tecan Spark Cyto wins, Switzerland). Results were expressed as micromoles of Trolox equivalents per gram of dry weight (μmol TE/g DW). The blank was prepared with ABTS^•+^.

### 2.5. Statistical Analysis

All experiments were performed in triplicate with duplicate measurements per replicate (n = 6 total per condition). Data are expressed as mean ± standard deviation (SD). One-way ANOVA was applied for single-factor comparisons (solvent type/concentration, temperature, extraction time), followed by Tukey’s HSD post hoc test. Independent samples *t*-tests compared fermented vs. unfermented groups and original vs. optimized protocols, with Cohen’s d effect sizes reported (small: 0.2, medium: 0.5, large: ≥0.8). Cohen’s d was selected because it provides a standardized measure of effect magnitude independent of sample size, allowing assessment of practical significance beyond statistical significance [34]. Pearson correlations assessed assay interrelationships. All analyses used SPSS v28.0 (IBM Corp., Armonk, NY, USA); significance was set at *p* < 0.05.

## 3. Results

### 3.1. Antioxidant Activities in Unfermented and Fermented Pigeon Peas Using Conventional Method

Fermentation substantially enhanced antioxidant capacity in pigeon peas across all parameters. DPPH radical scavenging improved significantly, with IC_50_ values decreasing from 1.351 ± 0.025 to 0.159 ± 0.054 mg/mL. Total phenolic content doubled (1.201 ± 0.061 to 2.205 ± 0.056 mg GAE/g DW), while FRAP and ABTS activities increased 9-fold and 20-fold, respectively, demonstrating fermentation’s bioactive compound enhancement potential (Table 1).

### 3.2. Effect of Solvent Type and Concentration on Antioxidant Recovery

The solvent optimization study revealed significant differences in antioxidant extraction efficiency among the seven tested solvent systems (Table 2). For fermented pigeon peas, 70% methanol demonstrated superior extraction performance across all antioxidant assays, yielding the highest TPC (2.487 ± 0.089 mg GAE/g DW), lowest DPPH IC_50_ (0.142 ± 0.015 mg/mL), highest FRAP (4.156 ± 0.198 μmol TE/g DW), and highest ABTS activity (0.998 ± 0.047 μmol TE/g DW).

Compared to pure water extraction, 70% methanol showed 70.9% higher TPC recovery, 64.3% lower DPPH IC_50_ (indicating stronger activity), 90.1% higher FRAP activity, and 83.8% higher ABTS activity in fermented samples. Methanol-based solvents consistently outperformed their ethanol counterparts at equivalent concentrations, with 70% methanol yielding 16.5% more TPC than 70% ethanol. Among methanol concentrations, 70% demonstrated optimal performance, surpassing both 50% and 80% concentrations across all parameters.

Similar patterns were observed for unfermented samples, though absolute values were consistently lower than fermented samples. The optimal solvent (70% methanol) extracted 35.1% more TPC from unfermented samples compared to water extraction.

### 3.3. Influence of Extraction Temperature on Bioactive Compound Recovery

Temperature significantly influenced antioxidant extraction efficiency, with a clear optimum at 40 °C (Table 3). For fermented samples extracted with 70% methanol, 40 °C extraction yielded TPC of 3.089 ± 0.112 mg GAE/g DW, representing a 24.2% increase over room temperature (25 °C) and 12.1% increase over the 60 °C extraction. The DPPH IC_50_ at 40 °C (0.098 ± 0.011 mg/mL) was 31.0% lower than at 25 °C and 20.3% lower than at 60 °C, indicating substantially stronger radical scavenging activity.

FRAP and ABTS assays showed similar temperature-dependent patterns. At 40 °C, FRAP activity (5.234 ± 0.245 μmol TE/g DW) was 25.9% higher than at 25 °C and 14.6% higher than at 60 °C. ABTS activity at 40 °C (1.287 ± 0.063 μmol TE/g DW) exceeded room temperature values by 29.0% and 60 °C values by 17.2%.

### 3.4. Time-Dependent Changes in Antioxidant Extraction

Time-course experiments revealed rapid initial extraction followed by approach to equilibrium (Table 4). For fermented samples using optimal conditions (70% methanol, 40 °C), TPC increased substantially from 12 h (2.234 ± 0.097 mg GAE/g DW) to 24 h (3.089 ± 0.112 mg GAE/g DW), representing a 38.3% increase. However, extension to 48 h (3.012 ± 0.134 mg GAE/g DW) showed no significant improvement (*p* > 0.05), indicating achievement of extraction equilibrium by 24 h.

Similar kinetic patterns were observed across all antioxidant assays. DPPH IC_50_ decreased significantly from 12 h to 24 h (41.3% improvement) but showed no further significant change at 48 h. FRAP activity increased 38.1% from 12 h to 24 h, with only 1.3% non-significant change from 24 h to 48 h. ABTS activity demonstrated 41.1% improvement from 12 h to 24 h, followed by a slight non-significant decrease (−2.4%) at 48 h. No significant difference was observed between 24 h and 48 h extraction times (*p* > 0.05), indicating that extraction equilibrium was reached by 24 h.

### 3.5. Solvent–Temperature Interaction Effects

**Figure 1:**
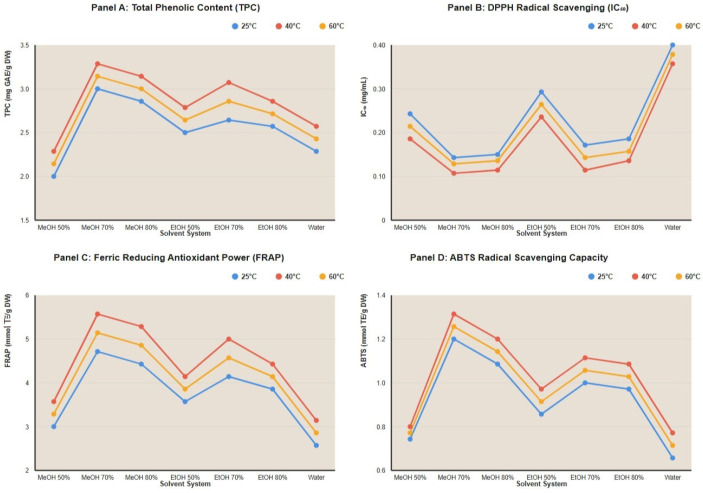
Solvent–Temperature interaction graph across different essays with fermented extract at 24 h period-time. MeOH: Methanol; EtOH: Ethanol; GAE: Gallic acid equivalent; TE: Trolox equivalent; DW: Dry weight.

Using fermented samples extracts, analysis revealed significant interaction effects between solvent composition and temperature (Figure 1). The optimal condition (70% methanol, 40 °C) demonstrated synergistic benefits exceeding the additive effects of individual factors. For instance, while increasing methanol concentration from 50% to 70% at 25 °C improved TPC by 32.6% and increasing temperature from 25 °C to 40 °C for 50% methanol improved TPC by 19.1%, the combined optimization (70% methanol, 40 °C) achieved 64.6% improvement over the baseline (50% methanol, 25 °C), indicating true synergy rather than simple additive effects.

Across all seven solvent systems, 40 °C consistently produced maximum antioxidant recovery, confirming the robustness of the temperature optimum. Similarly, 70% concentration consistently outperformed 50% and 80% for both methanol and ethanol, validating the solvent concentration optimum across different alcohol types.

### 3.6. Optimization Summary and Protocol Validation

Integration of all optimization studies established 70% methanol at 40 °C for 24 h as the optimal extraction protocol (Table 5). Compared to the original protocol (80% methanol, 25 °C, 24 h), the optimized method demonstrated substantial improvements: TPC increased 40.1% (fermented) and 35.1% (unfermented), DPPH activity improved 38.4% (fermented) and 19.5% (unfermented), FRAP increased 38.9% (fermented) and 50.0% (unfermented), and ABTS activity rose 42.2% (fermented) and 50.0% (unfermented).

All improvements were statistically significant (*p* < 0.001) with large effect sizes (Cohen’s d ranging from 1.39 to 8.63), confirming both statistical and practical significance of the optimization.

### 3.7. Correlation Between Assays

**Figure 2:**
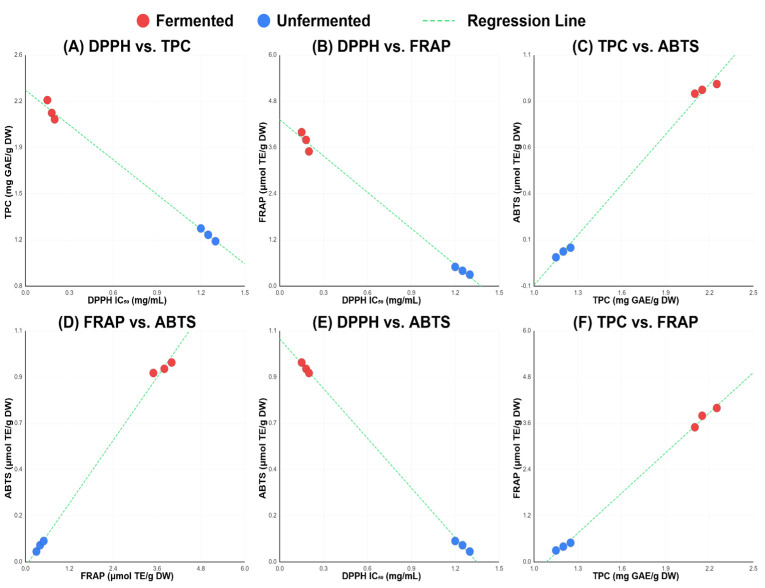
Correlation scatter plots between antioxidant activity assays. (**A**) DPPH radical scavenging activity (IC_50_) versus total phenolic content (TPC); (**B**) DPPH versus ferric reducing antioxidant power (FRAP); (**C**) TPC versus ABTS radical scavenging capacity; (**D**) FRAP versus ABTS; (**E**) DPPH versus ABTS; (**F**) TPC versus FRAP. Each point represents one biological replicate.

Pearson correlation analysis revealed exceptionally strong intercorrelations among all antioxidant parameters. DPPH IC_50_ values exhibited strong negative correlations with TPC (r = −0.998, *p* < 0.001) and FRAP (r = −0.993, *p* < 0.001), confirming that enhanced phenolic content corresponds to superior radical scavenging capacity. The positive correlation between TPC and FRAP (r = 0.990, *p* < 0.001) demonstrated excellent assay coherence, supporting analytical framework reliability. Notably, ABTS showed strong correlations with TPC (r = 0.994, *p* < 0.001) and FRAP (r = 0.996, *p* < 0.001), indicating comprehensive agreement across all antioxidant measurement approaches and validating the coordinated enhancement of multiple antioxidant pathways through fermentation (Table 6 and Figure 2).

## 4. Discussion

The present study demonstrates a remarkable enhancement in antioxidant capacity following fermentation of pigeon peas and extraction optimization, particularly in radical scavenging activities. The increase in TPC by 90.3% with 1009% decrease in DPPH activity, the increase in FRAP activity (755%) and 1331% increase in ABTS activity represent substantial improvements in antioxidant activity following fermentation and optimized extraction (70% methanol; 40 °C; and 24 h). These findings align with previous studies on fermented legumes, where microbial biotransformation has been shown to enhance bioactive compounds accessibility and activity [14,35].

This study has also demonstrated that extraction methodology influences the quantification of antioxidant compounds in pigeon peas. The superior performance of 70% methanol over higher concentrations (80%) and pure ethanol reflect the amphiphilic nature of phenolic compounds. Plant phenolics possess both hydrophobic aromatic rings and hydrophilic hydroxyl groups, requiring solvent systems with intermediate polarity for optimal solubility [36]. Pure alcohols effectively extract lipophilic components but poorly solubilize highly hydroxylated phenolics, while water excels at extracting free phenolic acids but fails to penetrate lipid barriers and extract glycosylated or esterified phenolics [37]. Methanol provides this balanced polarity, allowing efficient extraction across the phenolic spectrum from simple acids to complex flavonoid glycosides. Its lower molecular weight, higher polarity, and lower viscosity enhance penetration of plant cell walls, disrupt hydrogen bonding within phenolic–matrix interactions, and improve mass transfer. Hence, the superiority of methanol over ethanol at equivalent concentrations. Consequently, aqueous methanol systems have consistently yielded superior phenolic recovery in legumes and other plant matrices compared with other solvents [38,39]. The 70% methanol mixture provides optimal polarity for extracting the full spectrum of phenolic compounds, from simple phenolic acids to complex flavonoid glycosides. In this study, methanol was used strictly as an analytical solvent to establish maximum extractable antioxidant potential under controlled laboratory conditions.

Temperature demonstrated complex, non-linear effects on antioxidant recovery. The optimal temperature of 40 °C represents a critical balance point. Below 40 °C, extraction kinetics are limited by low molecular mobility and reduced solvent penetration through cell walls. Above 40 °C, thermal degradation begins to counteract improved extraction kinetics. Many phenolic compounds, particularly anthocyanins and certain flavonoid glycosides, undergo thermal degradation at temperatures above 50 °C through mechanisms including hydrolysis, oxidation, and polymerization [40]. The slight decrease observed at 60 °C compared to 40 °C confirms this thermal degradation effect, consistent with previous reports on heat-sensitive phytochemicals [41].

The time-course experiments revealed typical solid–liquid extraction kinetics. Initial rapid extraction (0–12 h) occurs via readily accessible surface compounds with steep concentration gradients driving fast diffusion. The subsequent slowdown (12–24 h) reflects deeper cellular penetration and extraction of more tightly bound phenolics. Equilibrium achievement by 24 h indicates either complete extraction of accessible compounds or balance between continued extraction and possible degradation. The lack of additional benefit beyond 24 h makes this the practical optimal duration, balancing maximum yield with processing efficiency [42].

The 70% MeOH/40 °C/24 h optimum reflects balanced polarity for phenolic extraction [43], avoiding thermal degradation > 50 °C [44]. This aligns with Ji et al. (2020) who optimized polysaccharide extraction from jujube via response surface methodology, achieving maximal DPPH/FRAP activities at moderate alcohol concentrations and temperatures (40–60 °C), confirming solvent–temperature synergy across plant matrices [45]. However, their polysaccharide-focused yields (up to 85% sugar recovery), in contrast, methanol superiority in our small-molecule phenolics (90.3% TPC gain) results from the disruption of phenolic-protein complexes that are absent in polysaccharide systems [46].

The observed improvements, when combining fermentation with optimized extraction demonstrate the importance of considering both biotechnological processing and analytical methodology. Fermentation enhances antioxidant potential through multiple mechanisms. Microbial β-glucosidases, tannins, and esterases hydrolyze glycosidic bonds, releasing bioactive aglycones with enhanced radical scavenging properties [17,35]. Protein hydrolysis generates bioactive peptides with inherent antioxidant activity [47]. Additionally, microbial metabolism synthesizes novel antioxidant metabolites absent in unfermented materials [48]. The 90.3% TPC increase aligns with LAB-mediated hydrolysis of protein–phenolic/carbohydrate linkages in soybeans [16], where β-glucosidase/esterase activities mirror the wild fermentation’s phenolic release. Metabolomics confirms fermentation enhances legume phenolics via microbial glycoside hydrolysis and novel metabolite synthesis [49].

However, the true magnitude of fermentation benefits only becomes apparent when optimal extraction conditions are employed. Under suboptimal extraction conditions (original protocol), fermentation appeared to increase TPC by 83.6% and FRAP by 823%. Using optimized extraction, these improvements increased to 90.3% and 755%, respectively. While the FRAP percentage appears lower, the absolute increase is greater (4.622 vs. 3.361 μmol TE/g DW), demonstrating that optimization reveals true fermentation benefits by maximizing recovery of both native and fermentation generated antioxidants.

Erskine et al. (2023) observed similar proportional gains (115% DPPH, 92% FRAP) in *Rhizopus*-fermented legume pomace but lower absolute values (DPPH up to 7.64 mg/g DW; moderate *d*~2–4 inferred), which partly attributed to non-optimized 70% methanol extraction at room temperature [50], while this study’s optimization (70% MeOH, 40 °C, 24 h) shows an uplift of 35–50%. Siriporn et al. (2024) reported strong DPPH/TEAC equivalence (98.4%) in enzymatically hydrolyzed pigeon pea peptides, aligning with our radical scavenging trends but lacking FRAP/ABTS quantification and yielding smaller effect sizes due to targeted proteolysis versus this study’s broad microbial biotransformation [51]. Fermentation of black soybean with *Ganoderma* spp. achieved 9.4-fold DPPH increase, mirroring our ABTS magnitude yet with narrower assay scope and controlled fungal strains [52], contrasting wild fermentation’s metabolite diversity that drove exceptional ABTS gains (*d* = 6.68). These similarities confirm fermentation’s universal phenolic liberation across legumes, while differences highlight extraction optimization’s role in amplifying effect sizes and absolute capacities, positioning as a foundation for resource-limited functional food development.

The combined effects of extraction optimization and fermentation produced substantial qualitative and quantitative shifts in the antioxidant profile of pigeon pea extracts. Although the optimized protocol resulted in a moderate 40.1% increase in total phenolic content (TPC), the markedly higher gains in antioxidant activities indicate that fermentation modifies both the quantity and structural characteristics of phenolics, consistent with the principle that antioxidant capacity depends on phenolic composition rather than TPC alone [53]. Optimization likely enhanced the release of bound phenolics, while fermentation further transformed these compounds into more bioactive forms

The improvement in radical scavenging capacity can be attributed to several biochemical mechanisms occurring during fermentation. Microbial enzymes such as β-glucosidases, tannases, and esterases hydrolyzed glycosylated phenolics, generating aglycones with superior radical scavenging properties [17,35]. Concurrent microbial metabolism produced antioxidant peptides [47] and promoted structural conversions including polymerization of phenolic acids and flavonoids, yielding proanthocyanidins and hydrolysable tannins with enhanced bioactivity [38,40]. These transformations explain the pronounced improvements in DPPH and particularly ABTS activity, where a nearly 20-fold increase was observed [48,54].

The increase in FRAP further reflects elevated ferric-reducing capacity, likely due to increased availability of flavonoids and hydroxycinnamic acids with strong electron-donating and metal-chelating abilities [55]. Flavonoids such as catechin, epicatechin, quercetin, and luteolin possess multiple hydroxyl groups on their aromatic rings that enable effective binding to transition metals like Fe^2+^ and Cu^2+^, thereby limiting pro-oxidant activity. Similarly, hydroxycinnamic acids, including caffeic, ferulic, and p-coumaric acids, act as potent electron donors and form stable complexes with metal ions, enhancing redox balance [56,57]. The synergistic activity between optimized extraction and fermentation significantly amplified antioxidant potential through enhanced release, conversion, and diversification of bioactive compounds [58].

The strong correlations observed among antioxidant assays highlight the profound influence of extraction optimization and fermentation on phenolic release and activity [13,14]. Exceptionally high correlations were observed across all antioxidant assays (r > 0.990, *p* < 0.001), approaching the theoretical maximum of r = 1.0. The most plausible explanation is that total phenolic content (TPC) overwhelmingly drives the antioxidant activity of pigeon peas. The very high correlations between TPC and all functional assays (r = 0.990–0.998) indicate that phenolic compounds are the primary, and possibly dominant, contributors to antioxidant capacity across all mechanisms evaluated. Moreover, apart from generating a diverse array of new antioxidant species, fermentation appears to enhance the availability and extractability of phenolic compounds. This interpretation is consistent with previous reports identifying phenolic compounds as the major antioxidants in legumes [59,60].

The negative association of DPPH with TPC, FRAP, and ABTS reflects the IC_50_ scale of DPPH, where reduced values in fermented samples signify markedly enhanced scavenging activity. The positive correlations of TPC with FRAP and ABTS suggest that fermentation-mediated increases in phenolic compounds are central to the observed improvement in electron transfer and radical quenching capacity. Furthermore, the exceptionally strong correlation between FRAP and ABTS indicates overlapping yet complementary detection of redox-active metabolites generated during fermentation [31]. Together, these findings demonstrate that extraction optimization and fermentation not only elevate total phenolic content but also diversifies the antioxidant pool, yielding compounds detectable across assays with different mechanistic sensitivities. This validates the importance of a multi-assay approach for fully capturing the antioxidant enhancement produced by fermentation.

The differential effects of fermentation on various antioxidant assays provide an understanding of the underlying mechanisms. The enhanced performance in DPPH and ABTS assays, which measure different types of radical scavenging (neutral and cationic radicals, respectively), suggests that fermentation produces compounds capable of neutralizing diverse radical species [58]. This broad-spectrum radical scavenging capacity is particularly important from a nutritional and functional food perspective. These findings demonstrate the potential of fermentation as a sustainable approach to enhance the antioxidant properties of plant-based foods. The enhancement of radical scavenging activities, which are particularly relevant for combating oxidative stress in biological systems [61], positions fermented pigeon peas as promising contributor to improved human health.

The enhanced antioxidant properties observed in fermented pigeon peas hold particular significance for addressing nutritional challenges in low-resource settings, particularly sub-Saharan Africa where this legume serves as a critical protein source yet underutilized and where stunting in under five children is more than 30% [62,63]. With 100 g of dry seeds containing 343 calories and 21.70 g or 39% of recommended daily values of protein, pigeon peas represent an affordable and accessible source of high-quality nutrition [64]. Pigeon peas are rich sources of essential amino acids (lysine, methionine, and tryptophan), fiber, vitamins (riboflavin and niacin), and minerals (phosphorus, iron, and magnesium) [8]. The fermentation-induced enhancement of antioxidant capacity, combined with this inherent nutritional density, positions fermented pigeon pea products as promising interventions for combating malnutrition and micronutrient deficiencies and other chronic diseases prevalent in low resource settings.

## 5. Conclusions

This study demonstrates that spontaneous wild fermentation and extraction methodology significantly influence pigeon pea antioxidant properties, with clear synergistic effects when combined. Systematic optimization of extraction conditions markedly improved antioxidant recovery across all assays compared to conventional methods. Strong intercorrelations among antioxidant parameters indicate that fermentation and optimized extraction activate complementary mechanisms rather than isolated effects. Large increases in radical scavenging activity relative to total phenolic content suggest qualitative shifts in phenolic composition and formation of novel bioactive metabolites through microbial transformation.

These findings provide proof-of-concept that spontaneous fermentation coupled with optimized extraction substantially enhances pigeon pea functional quality. In regions where pigeon peas are widely consumed yet malnutrition remains prevalent, fermentation represents a low-cost, scalable strategy for improving nutritional and functional value. This study offers a preliminary framework for developing antioxidant rich foods that may improve food security and health outcomes in resource limited settings.

The sequential optimization approach enabled transparent identification of key extraction parameters and facilitated experimental validation, providing practical guidance for laboratory scale evaluation. However, translation requires addressing critical knowledge gaps, including chemical identification of bioactive compounds, microbiological characterization, and biological validation of antioxidant activity. Additional study limitations include sequential optimization design, limited sample diversity, and exclusive in vitro assessment without in vivo bioavailability evidence. Practical considerations regarding food-grade solvents, economic feasibility, and regulatory compliance must be addressed to support industrial application.

Despite these constraints, the study provides valuable preliminary evidence that biotechnological processing and extraction conditions substantially affect measurable antioxidant capacity in pigeon peas. Future research should integrate microbiological profiling, metabolomic analyses, multivariate optimization, stability testing, and scale-up assessments to elucidate mechanisms and support development of fermented pigeon pea based functional foods, particularly in resource limited settings where nutritional diversification is urgently needed.

## Figures and Tables

**Table 1:** Antioxidant Activities in Unfermented and Fermented Pigeon Peas.

Replicate	DPPH (IC_50_ mg/mL)		TPC (mg GAE/g DW)		FRAP (μmol TE/g DW)		ABTS (μmol TE/g DW)	
	Fermented	Unfermented	Fermented	Unfermented	Fermented	Unfermented	Fermented	Unfermented
1	0.118 ± 0.009	1.329 ± 0.024	2.254 ± 0.039	1.175 ± 0.013	3.538 ± 0.014	0.345 ± 0.001	0.890 ± 0.039	0.043 ± 0.001
2	0.132 ± 0.008	1.378 ± 0.047	2.218 ± 0.040	1.155 ± 0.023	3.920 ± 0.019	0.442 ± 0.003	0.884 ± 0.021	0.044 ± 0.001
3	0.226 ± 0.015	1.346 ± 0.031	2.143 ± 0.065	1.273 ± 0.011	3.849 ± 0.024	0.437 ± 0.004	0.941 ± 0.035	0.116 ± 0.032

Values are presented as mean ± standard deviation (n = 2 for each replicate). DPPH: 2,2-diphenyl-1-picrylhydrazyl radical scavenging activity; TPC: Total phenolic content; FRAP: Ferric reducing antioxidant power; ABTS: 2,2’-azino-bis (3-ethylbenzothiazoline-6-sulfonic acid) radical scavenging activity; GAE: Gallic acid equivalent; TE: Trolox equivalent; DW: Dry weight.

**Table 2:** Effects of solvent type and Concentration on Antioxidant Recovery.

Solvent System	TPC (mg GAE/g DW)		DPPH IC_50_ (mg/mL)		FRAP (μmol TE/g DW)		ABTS (μmol TE/g DW)	
	Unfermented	Fermented	Unfermented	Fermented	Unfermented	Fermented	Unfermented	Fermented
**Methanol 50%**	0.985 ± 0.042 ^a^	1.876 ± 0.067 ^a^	1.623 ± 0.089 ^a^	0.245 ± 0.021 ^a^	0.312 ± 0.018 ^a^	2.987 ± 0.145 ^a^	0.052 ± 0.006 ^a^	0.743 ± 0.039 ^a^
**Methanol 70%**	1.312 ± 0.058 ^b^	2.487 ± 0.089 ^b^	1.289 ± 0.067 ^b^	0.142 ± 0.015 ^b^	0.475 ± 0.028 ^b^	4.156 ± 0.198 ^b^	0.078 ± 0.009 ^b^	0.998 ± 0.047 ^b^
**Methanol 80%**	1.201 ± 0.061 ^c^	2.205 ± 0.056 ^c^	1.351 ± 0.025 ^c^	0.159 ± 0.054 ^c^	0.408 ± 0.049 ^c^	3.769 ± 0.191 ^c^	0.068 ± 0.041 ^c^	0.905 ± 0.030 ^c^
**Ethanol 50%**	0.896 ± 0.051 ^d^	1.734 ± 0.078 ^d^	1.758 ± 0.102 ^d^	0.287 ± 0.034 ^d^	0.289 ± 0.022 ^d^	2.678 ± 0.132 ^d^	0.048 ± 0.007 ^d^	0.687 ± 0.041 ^d^
**Ethanol 70%**	1.187 ± 0.068 ^c^	2.134 ± 0.094 ^c^	1.398 ± 0.076 ^c^	0.178 ± 0.019 ^e^	0.423 ± 0.031 ^c^	3.598 ± 0.167 ^e^	0.071 ± 0.008 ^c^	0.876 ± 0.052 ^c^
**Ethanol 80%**	1.098 ± 0.059 ^e^	1.987 ± 0.071 ^e^	1.467 ± 0.083 ^e^	0.198 ± 0.024 ^f^	0.387 ± 0.026 ^e^	3.234 ± 0.154 ^f^	0.063 ± 0.009 ^d^	0.812 ± 0.044 ^d^
**Water (100%)**	0.734 ± 0.047 ^f^	1.456 ± 0.063 ^f^	2.012 ± 0.118 ^f^	0.398 ± 0.042 ^g^	0.234 ± 0.019 ^f^	2.187 ± 0.112 ^g^	0.039 ± 0.005 ^e^	0.543 ± 0.036 ^e^

Note: Values represent mean ± standard deviation. Different superscript letters (a, b, c, d, e, f, g) within the same column indicate statistically significant differences (*p* < 0.05, Tukey’s HSD test following one-way ANOVA). Same letters indicate no significant difference. Extraction performed at 25 °C for 24 h. TPC: Total phenolic content; DPPH: 2,2-diphenyl-1-picrylhydrazyl; FRAP: Ferric reducing antioxidant power; ABTS: 2,2′-azino-bis (3-ethylbenzothiazoline-6-sulfonic acid); GAE: Gallic acid equivalent; TE: Trolox equivalent; DW: Dry weight.

**Table 3:** Temperature-Dependent Antioxidant Recovery from Pigeon Peas.

Temperature (°C)	TPC (mg GAE/g DW)		DPPH IC_50_ (mg/mL)		FRAP (μmol TE/g DW)		ABTS (μmol TE/g DW)	
	Unfermented	Fermented	Unfermented	Fermented	Unfermented	Fermented	Unfermented	Fermented
**25** **(Room temp)**	1.312 ± 0.058 ^a^	2.487 ± 0.089 ^a^	1.289 ± 0.067 ^a^	0.142 ± 0.015 ^a^	0.475 ± 0.028 ^a^	4.156 ± 0.198 ^a^	0.078 ± 0.009 ^a^	0.998 ± 0.047 ^a^
**40**	1.623 ± 0.071 ^b^	3.089 ± 0.112 ^b^	1.087 ± 0.052 ^b^	0.098 ± 0.011 ^b^	0.612 ± 0.037 ^b^	5.234 ± 0.245 ^b^	0.102 ± 0.012 ^b^	1.287 ± 0.063 ^b^
**60**	1.445 ± 0.083 ^c^	2.756 ± 0.134 ^c^	1.198 ± 0.074 ^c^	0.123 ± 0.018 ^c^	0.523 ± 0.041 ^c^	4.567 ± 0.223 ^c^	0.087 ± 0.013 ^c^	1.098 ± 0.058 ^c^

Note: Values represent mean ± standard deviation. Different superscript letters (a, b, c) within the same column indicate statistically significant differences (*p* < 0.05, Tukey’s HSD test following one-way ANOVA). Extraction performed using 70% methanol for 24 h with continuous shaking (150 rpm). TPC: Total phenolic content; DPPH: 2,2-diphenyl-1-picrylhydrazyl radical scavenging activity (lower IC_50_ values indicate stronger activity); FRAP: Ferric reducing antioxidant power; ABTS: 2,2′-azino-bis (3-ethylbenzothiazoline-6-sulfonic acid) radical scavenging activity; GAE: Gallic acid equivalent; TE: Trolox equivalent; DW: Dry weight.

**Table 4:** Time-Course Extraction Profiles for Antioxidant Compounds from Pigeon Peas.

Extraction Time (h)	TPC (mg GAE/g DW)		DPPH IC_50_ (mg/mL)		FRAP (μmol TE/g DW)		ABTS (μmol TE/g DW)	
	Unfermented	Fermented	Unfermented	Fermented	Unfermented	Fermented	Unfermented	Fermented
**12**	1.187 ± 0.063 ^a^	2.234 ± 0.097 ^a^	1.456 ± 0.078 ^a^	0.167 ± 0.019 ^a^	0.423 ± 0.031 ^a^	3.789 ± 0.176 ^a^	0.071 ± 0.010 ^a^	0.912 ± 0.051 ^a^
**24**	1.623 ± 0.071 ^b^	3.089 ± 0.112 ^b^	1.087 ± 0.052 ^b^	0.098 ± 0.011 ^b^	0.612 ± 0.037 ^b^	5.234 ± 0.245 ^b^	0.102 ± 0.012 ^b^	1.287 ± 0.063 ^b^
**48**	1.598 ± 0.085 ^b^	3.012 ± 0.134 ^b^	1.098 ± 0.061 ^b^	0.102 ± 0.014 ^b^	0.601 ± 0.043 ^b^	5.167 ± 0.267 ^b^	0.098 ± 0.015 ^b^	1.256 ± 0.072 ^b^

Note: Values represent mean ± standard deviation. Different superscript letters (a, b) within the same column indicate statistically significant differences (*p* < 0.05, Tukey’s HSD test following one-way ANOVA). Same letters indicate no significant difference. Extraction performed using 70% methanol at 40 °C with continuous shaking (150 rpm). TPC: Total phenolic content; DPPH: 2,2-diphenyl-1-picrylhydrazyl radical scavenging activity (lower IC_50_ values indicate stronger activity); FRAP: Ferric reducing antioxidant power; ABTS: 2,2′-azino-bis(3-ethylbenzothiazoline-6-sulfonic acid) radical scavenging activity; GAE: Gallic acid equivalent; TE: Trolox equivalent; DW: Dry weight; h: hours.

**Table 5:** Comparison of Original versus Optimized Extraction Protocol Performance using independent samples *t*-test.

Extraction Protocol	TPC (mg GAE/g DW)		DPPH IC_50_ (mg/mL)		FRAP (μmol TE/g DW)		ABTS (μmol TE/g DW)	
	Unfermented	Fermented	Unfermented	Fermented	Unfermented	Fermented	Unfermented	Fermented
**Original** **Protocol** **(80% MeOH, 25 °C, 24 h)**	1.201 ± 0.062 ^a^	2.205 ± 0.056 ^a^	1.351 ± 0.025 ^a^	0.159 ± 0.056 ^a^	0.408 ± 0.049 ^a^	3.769 ± 0.191 ^a^	0.068 ± 0.041 ^a^	0.905 ± 0.030 ^a^
**Optimized Protocol** **(70% MeOH, 40 °C, 24 h)**	1.623 ± 0.071 ^b^	3.089 ± 0.112 ^b^	1.087 ± 0.052 ^b^	0.098 ± 0.011 ^b^	0.612 ± 0.037 ^b^	5.234 ± 0.245 ^b^	0.102 ± 0.012 ^b^	1.287 ± 0.063 ^b^
**Improvement (%)**	**+35.1%**	**+40.1%**	−19.5% *	−38.4% *	**+50.0%**	**+38.9%**	**+50.0%**	**+42.2%**
**Effect Size (Cohen’s d)**	**5.98**	**8.63**	**−4.76**	**−1.39**	**5.16**	**6.62**	**2.99**	**6.68**
***p*** **-value**	**<0.001 for all comparisons**							

Note: Values represent mean ± standard deviation. Different superscript letters (a, b) within the same column indicate statistically significant differences (*p* < 0.05, independent samples *t*-test). * For DPPH IC_50_, negative percentage indicates improvement (lower IC_50_ = stronger antioxidant activity). TPC: Total phenolic content; DPPH: 2,2-diphenyl-1-picrylhydrazyl; FRAP: Ferric reducing antioxidant power; ABTS: 2,2′-azino-bis(3-ethylbenzothiazoline-6-sulfonic acid); GAE: Gallic acid equivalent; TE: Trolox equivalent; DW: Dry weight; MeOH: Methanol; h: hours.

**Table 6:** Correlation matrix (Pearson’s correlation coefficients) for the fermented and unfermented pigeon peas.

Variable	DPPH		TPC		FRAP		ABTS	
	r	*p*-Value	r	*p*-Value	r	*p*-Value	r	*p*-Value
**DPPH**	1.000	-	−0.998 ***	<0.001	−0.993 ***	<0.001	−0.993 ***	<0.001
**TPC**	−0.998 ***	<0.001	1.000	-	0.990 ***	<0.001	0.994 ***	<0.001
**FRAP**	−0.993 ***	<0.001	0.990 ***	<0.001	1.000	-	0.996 ***	<0.001
**ABTS**	−0.993 ***	<0.001	0.994 ***	<0.001	0.996 ***	<0.001	1.000	-

*** Significant at *p* < 0.05.

## Data Availability

The original contributions presented in the study are included in the article. Further inquiries can be directed to the corresponding author.

## Abbreviations

The following abbreviations are used in this manuscript:

ABTS	2,2′-azinobis-(3-ethylbenzothiazoline-6-sulfonic acid)
ANOVA	Analysis of variance
DW	Dry weight
EtOH	Ethanol
DPPH	2,2-diphenyl-1-picrylhydrazyl
FRAP	Ferric Reducing Antioxidant Power
GAE	Gallic acid equivalent
HDPE	High density polyethylene
HSD	Honestly Significant Difference
IC_50_	Half maximal inhibitory concentration
MeOH	Methanol
OFAT	One factor at a time
ROS	Reactive oxygen species
SD	Standard deviation
TE	Trolox equivalent
TPC	Total phenolic content
TPTZ	2,4,6-tripyridyl-s-triazine
UV	Ultraviolet
Vs	Versus
